# Management of lenticule detachment and epithelial downgrowth after Descemet stripping automated endothelial keratoplasty: a novel technique and brief literature review

**DOI:** 10.1186/s12886-022-02264-7

**Published:** 2022-01-29

**Authors:** Mohammad Soleimani, Arash Alizadeh, Maziyar Irannejad, Mansoor Shahriari, Kasra Cheraqpour

**Affiliations:** 1 Eye Research Center, Farabi Eye Hospital, Tehran University of Medical Sciences, Tehran, Iran; 2 Imam Hossein Medical Center, Shahid Beheshti University of Medical Sciences, Tehran, Iran

**Keywords:** Epithelial downgrowth, Descemet stripping automated endothelial Keratoplasty, DSAEK, Surgical technique, Keratoplasty

## Abstract

**Background:**

Epithelial downgrowth is a rare complication after Descemet stripping automated endothelial keratoplasty (DSAEK), which usually leads to poor visual outcome despite multiple available options of treatment.

**Case presentation:**

A 50-year-old man underwent DSAEK procedure due to pseudophakic bullous keratopathy. Three months later, the patient presented with gradual visual loss; slit-lamp examination revealed detachment and folding of the DSAEK lenticule, which was confirmed by anterior segment optical coherence tomography. On confocal scanning, epithelial cell sheets were detected in the interface leading to the wrinkling of the donor tissue and donor detachment. Surgical debridement and transient fixating with straight 10–0 prolene needles were performed followed by air injection into the anterior chamber. The cornea turned clear in the one-year follow-up with uncorrected-visual acuity of 20/30 and best-corrected visual acuity of 20/25.

**Conclusions:**

Early diagnosis and treatment of epithelial downgrowth may be associated with a good prognosis and prevent from more aggressive treatments such as repeat of grafting. In this case, mechanical debridement and transient fixation of lenticule by 10–0 prolene needles was performed to manage post-DSAEK epithelial downgrowth and lenticule detachment, which was successful without requiring of additional re-grafting. It seems this is a feasible technique with acceptable long-term outcomes.

**Supplementary Information:**

The online version contains supplementary material available at 10.1186/s12886-022-02264-7.

## Background

Penetrating injuries and intraocular surgeries may be complicated with invasion of the epithelium into the anterior chamber, which is called epithelial downgrowth (ED) or ingrowth [1]. Mechanism of ED is complex and multiple risk factors such as poor wound closure, numerous intraocular surgeries, wound fistula, and iris or vitreous incarceration may have a role. ED manifests as milky sheets onto the posterior surface of the cornea with possible extension to the iris and anterior chamber angle [2]. Severe cases of this condition can lead to corneal endothelial failure, corectopia, and refractory glaucoma. The incidence of ED after cataract surgery and PK is about 0.1% and 0.25%, respectively [1]. Management of this potentially vision-threatening entity is so challenging and the prognosis is guarded [3].

To the best of the author’s knowledge and database search, few cases of ED after DSAEK have been reported in the last decades. We report a case of lenticule detachment and epithelial downgrowth after Descemet Stripping Automated Endothelial Keratoplasty, which managed through surgical debridement and transient fixating of lenticule by straight 10–0 prolene needles not requiring a re-grafting.

## Case presentation

**Fig. 1:**
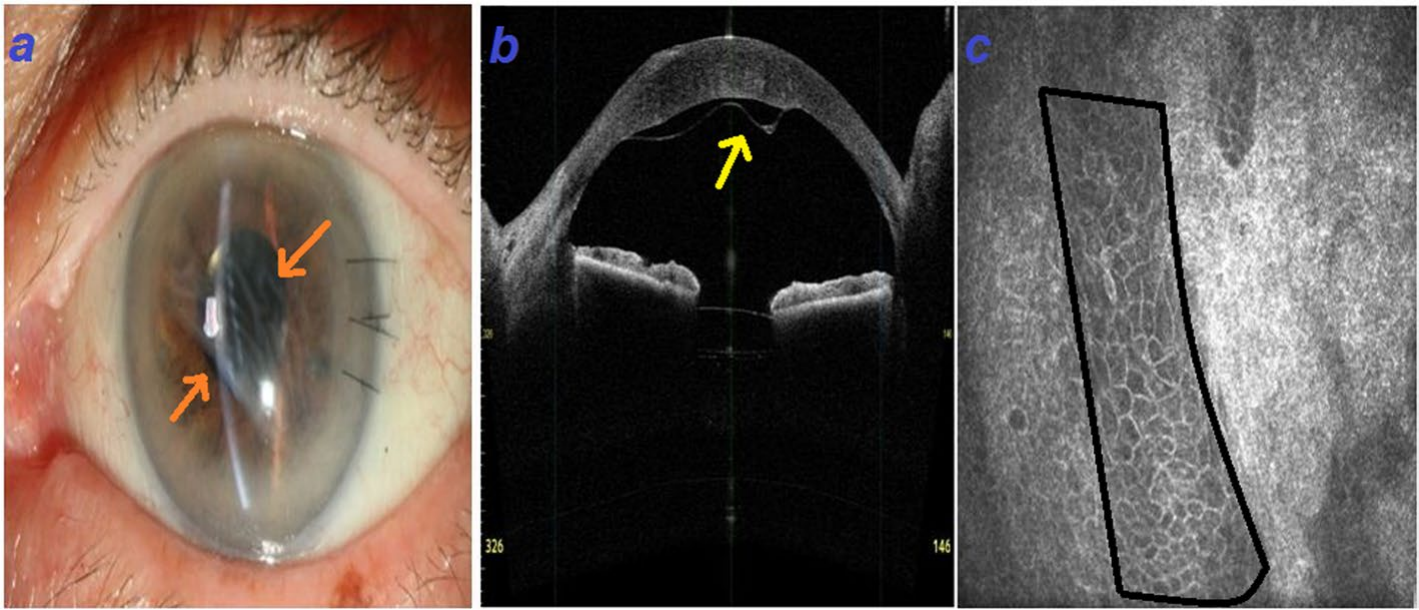
Note to the white sheets on the endothelial surface representing lenticule detachment and epithelial downgrowth (orange arrows) (**a**). Anterior segment optical coherence tomography shows retraction and folding of the graft (yellow arrow) (**b**), Confocal scanning documented sheets of epithelial cells trapped in the stroma (marked area in black borders) (**c**)

A 50-year-old man presented with pseudophakic bullous keratopathy after cataract surgery. His visual acuity was counting fingers at 1 m; the posterior capsule was intact with well-positioned intraocular lens (IOL) in the bag. The patient was scheduled for DSAEK surgery. In the DSAEK procedure, recipient corneal epithelium was removed and four venting incisions were made in the midperiphery of the cornea before Descemet stripping. The donor cornea was inserted using a Busin glide; after DSAEK lenticlue insertion, air injection was done up to the border of DSAEK lenticule. The surgical procedure resulted in a clear cornea. However, the patient presented with gradual visual loss three months later and the DSAEK lenticule was detached, non-mobile and folded confirmed by anterior segment optical coherence tomography (ASOCT) (CASIA SS-1000, TOMEY, Nagoya, Japan) (Fig. 1a, b). On confocal scanning microscopy (Heidelberg Retina Tomograph 3 with the Rostock Cornea Module, Heidelberg Engineering GmbH, Dossenheim, Germany), epithelial cell sheets were detected in the interface leading to the wrinkling of the donor tissue and detachment (Fig. 1c).

**Fig. 2:**
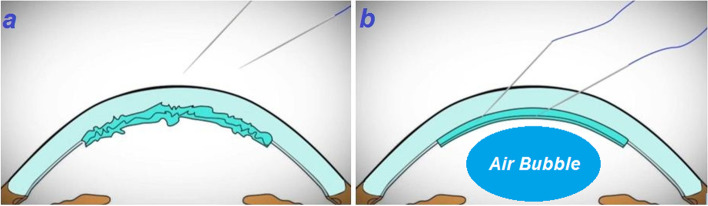
Wrinkling of the donor tissue and donor detachment at the presentation (**a**). Using several 10–0 prolene needles for transient fixation of lenticule (**b**)

**Fig. 3:**
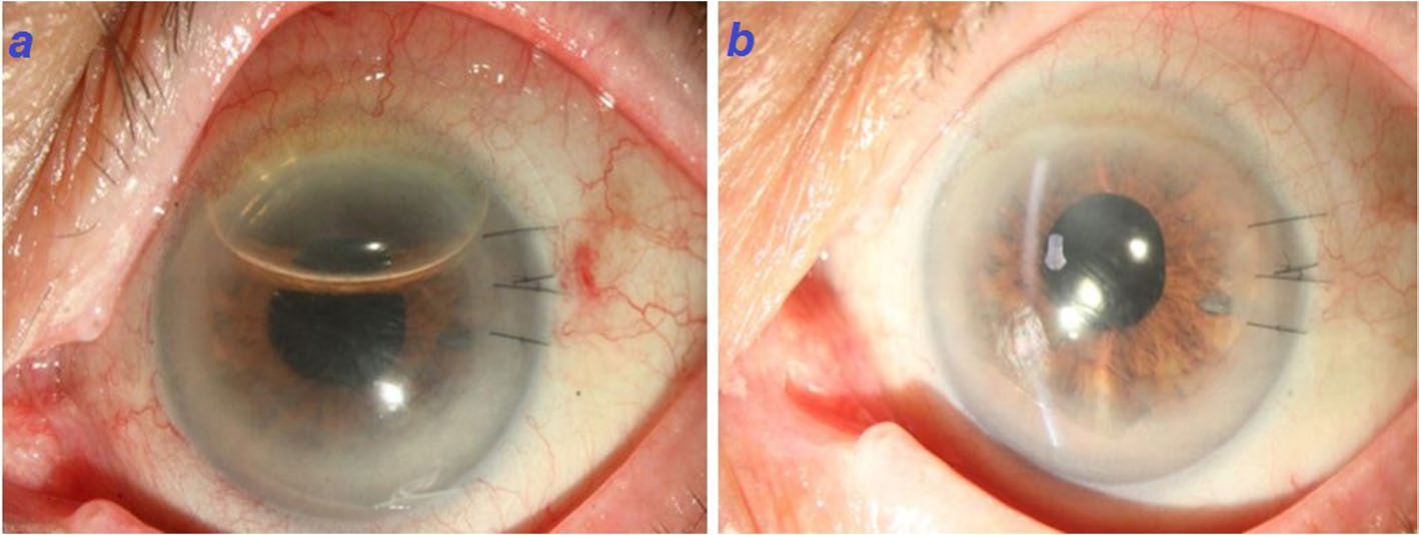
First day follow-up visit after surgical debridement and air injection (**a**). Second follow-up visit after surgical procedure which shows a clear graft after resolution of epithelial downgrowth (**b**)

After general anesthesia, proper prepping and draping was done followed by placement of eyelid speculum and irrigation of ocular surface by povidone-iodine. In the primary maneuvers refractory nature of the detached lenticule to repositioning was noted and despite multiple attempts for re-surfacing and attachment of lenticule, shrinkage was happened. We thought that entered epithelial cells from venting incisions were culprit for this event. So, from the venting incision, epithelial cell sheets of the interface, which were diagnosed on confocal scanning and AS-OCT were carefully debrided and scraped using a secondary instrument and double cannula. After repeated irrigation and aspiration, epithelial strands were removed as much as possible. However, possibility of remaining some epithelial cells is still there. Then, we used several straight 10–0 prolene needles for transient fixation of lenticule followed by full air injection (Fig. 2a, b). The needles were removed after five minutes and the air was deflated up to the lenticule border. These actions led to unfolded lenticule. The cornea turned clear in the one-year follow-up, with uncorrected-visual acuity of 20/30 and best-corrected visual acuity of 20/25 (Fig. 3a, b) (Video 1). Also, preoperative and postoperative (1st year follow-up visit) CCT were 928 μm and 598 μm, respectively.

## Discussion and conclusions

Epithelial downgrowth is an uncommon condition. It may lead to devastating outcomes and catastrophic complications. Various intraocular surgeries such as cataract surgery, penetrating keratoplasty (PK), glaucoma filtering surgery, Descemet membrane endothelial keratoplasty (DMEK), and DSAEK may be complicated with ED [1]. It seems that outcomes of ED after DSAEK are probably better than non-DSAEK cases [4].

Previous studies have been shown that different risk factors such as several intraocular operations, poor wound closure, iris or vitreous incarceration, long-standing inflammation, corneal vascularization, and low intraocular pressure (IOP) may prone the eye to ED [1]. Moreover, the presence of intact Descemet membrane and endothelium is critical to block migration of epithelium into the anterior chamber, which can be lost due to DSAEK procedure. In other words, probable graft detachment may make a scaffold for the invasion of epithelial cells [5]. Risk factors of our patient were epithelial debridement during the procedure, large venting incision, use of 4 venting incisions, and also prolonged usage of contact lens which could transmit epithelial cells into the interface space to form a nest for subsequent retraction and donor tissue detachment.

Subtypes of ED include epithelial sheets, pearls, and cysts. Sheet type is the most common type of ED as occurred in our patient. Also, this type is the most destructive type, which may cause vision loss through glaucoma and corneal endothelial failure [1]. One study has been hypothesized that dragging of epithelial cells into the anterior chamber, decentered trephination of the graft, and implantation of epithelial cells from full-thickness corneal incision may play a role in pathophysiology of ED after DSAEK [1]. Epithelial cells may originate from either recipient or donor tissues [6]. In our case, several sources may be associated with the beginning location of epithelial downgrowth including the primary wound, paracentesis incisions and venting incisions. We had been used from 4 venting incisions during surgery including: supratemporal, supranasal, infratemporal, and infranasal. Figure 1a is showing the epithelial sheets which are arranged from supranasal to infratemporal compatible with location of our venting incisions. Hence, it seems inculpating venting incision for source of downgrowth is not irrational. However, patient’s corneal arcus has masked the changes of peripheral cornea in the images.

These patients usually turn symptomatic with decreased vision or pain. Non-specific features such as a hazy cornea, inflammation, and high IOP may make the diagnosis difficult. So, clinical suspicious can be helpful in these situations, especially in cases with history of previous ocular surgery. ED must be differentiated from iridocorneal endothelial (ICE) syndrome and metastatic carcinoma [7].

The gold standard method of the diagnosis is a histological assessment, which can also differentiate between the corneal or conjunctival origin of ED through the presence of goblet cells in cases with conjunctival source. Also, argon laser photocoagulation has a diagnostic value, which shows whitening of burn spots on invaded iris surface whereas healthy areas appear dark. Other noninvasive diagnostic modalities include ASOCT, confocal scanning, and specular microscopy [2].

Although there are some reports of spontaneous resolution of ED, various treatment options including surgical removal, cryotherapy, wide excision with adjunctive argon laser ablation, re-grafting through PK, DMEK, or DSAEK, and injection of antimetabolites like 5-FU into the anterior chamber are available. However, the failure rate of treatment is remarkable. It could be mentioned use of 5-FU was not successful in some cases [2]. Information on some of recent reports of ED after DSAEK (with available full text which managed surgically) including the elapsed time after procedure, the type of treatment, and visual outcomes are listed in Table 1.

**Table 1:** Information of some of recent reports of epithelial downgrowth after DSAEK

No.	Authors	Elapsed time after DSAEK	Risk factors	Method of diagnosis	Type of treatment	Using of 5-FU	Visual outcome
1	Gorovoy et al. [6]	4 years	Decentered trephination	Histopathological evaluation	Repeat of DSAEK	No	Initial BCVA: 20/30 Final BCVA: 20/30
2	Itty et al. [4]	7 months	Eccentric trephination	Histopathological examination	Ablation with diode laser which was not successful and lead to more surgeries including PK.	Yes	Initial BCVA: Not mentioned Final pinhole vision: 20/200
		32 months	Unknown	Histopathological examination	Argon laser ablation, surgical membranectomy with repeat of DSAEK	Yes	Initial BCVA: Not mentioned Final BCVA: 20/60
		32 months	Several intraocular surgeries	Histopathological examination	Diode laser Ablation, repeat of DSAEK	Yes	Initial BCVA: Not mentioned Final BCVA: Not mentioned
3	Phillips et al. [8]	6 months	Multiple surgeries, presence of vitreous within the surgical wound	Histopathological examination	Repeat of DSAEK	No	Initial BCVA: 20/400 Final BCVA: 20/80
4	Prasher et al. [5]	15 months	significant postoperative inflammation, multiple surgeries, detached graft	Histopathological examination, ASOCT	PK	No	Initial BCVA: 20/200 Final BCVA: Not mentioned
		14 months	significant postoperative inflammation, multiple surgeries	Histopathological examination, ASOCT	Repeat of DSAEK	No	Initial BCVA: 20/400 Final BCVA: Not mentioned
5	Wong et al. [9]	7 months	Multiple intraocular surgeries	Argon laser photocoagulation, histopathological examination	Repeat DSAEK	Yes	Initial BCVA: 20/100 Final BCVA: 20/30
6	Walker et al. [10]	3 months	Multiple intraocular surgeries, prolonged inflammation	In Vivo Confocal Microscopy, histopathological examination	PK with adjunctive cryotherapy	No	Initial pinhole vision: 20/80 Final BCVA: Not mentioned

The unique aspect of our report is management of epithelial downgrowth without requirement to re-grafting. Unlike previous reports of epithelial downgrowth after DSAEK which led to re-grafting (re-DSAEK or PK) after a short duration despite primary management with debridement or using 5-FU, our patient is currently stable through our novel technique without re-grafting (one-year follow-up, with uncorrected-visual acuity of 20/30 and best-corrected visual acuity of 20/25). It should be mentioned that the presence of epithelial sheets and diagnosis of ED was made using confocal scanning since unlike the typical cases of epithelial downgrowth, epithelial sheets were so fine in our complicated case leading to poor visibility through surgical microscope. It’s of note access and cleaning of epithelial sheets was done from venting incisions and interface scraping (but not excision) was tried to decrease the epithelial downgroth as less as possible. The authors have no claim regarding complete removal of epithelial sheets since this is not rationally possible and we think the reason of refractory nature of lenticule to repositioning is presence of remnant epithelial sheets. In this condition use of 10–0 prolene needles beside air injection was helpful in stretching of lenticule resulting in attachment. The concern of further endothelial damage by penetration of lenticule is logically right but it should be mentioned the needles have very tiny cross section and we believe endothelial damage following possible penetration was not significant. Our witness for this claim is comparison between preoperative and postoperative endothelial cell count (at 1st year of follow-up) in which 2327 cells/mm^2^ was changed to 2109 cells/mm^2^.

At the time being, no study is available on direct comparison between outcomes of graft exchanging and debridement. Although this condition is so rare, future studies may be helpful to address the preferred option. Nevertheless, we deeply believe that giving a chance to the patients for prevention from re-grafting and its subsequent issues is a logical approach.

In conclusion, early diagnosis and treatment of epithelial downgrowth may be associated with a good prognosis and prevent from more aggressive treatments such as repeat of grafting. In this case, mechanical debridement and transient fixation of lenticule by 10–0 prolene needles was performed to manage post-DSAEK epithelial downgrowth and lenticule detachment, which was successful without requiring of additional re-grafting. It seems this is a feasible technique with acceptable long-term outcomes.

## Supplementary Information

**Additional file 1: Video 1.** Shows the used surgical technique.

## Data Availability

The data is available from the corresponding author on reasonable request.

## Abbreviations

ED: epithelial downgrowth
PK: penetrating keratoplasty
DSAEK: Descemet stripping automated endothelial keratoplasty
IOL: intraocular lens
ASOCT: anterior segment optical coherence tomography
DMEK: Descemet membrane endothelial keratoplasty
IOP: intraocular pressure
ICE: iridocorneal endothelial syndrome
